# Evaluation of Maternal Risk Factors for Neonatal Hypernatremic Dehydration: A Systematic Review

**DOI:** 10.34763/jmotherandchild.20232701.d-24-00007

**Published:** 2024-08-06

**Authors:** Zakerihamidi Maryam, Rakhshanizadeh Forough, Moradi Ali, Ramezani Asal, Boskabadi Hassan

**Affiliations:** Department of Midwifery, School of Medical Sciences, Tonekabon Branch, Islamic Azad University, Tonekabon, Iran; Department of Pediatrics, Faculty of Medicine, Mashhad University of Medical Sciences, Mashhad, Iran; Clinical Research Development Unit, Ghaem Hospital, Mashhad University of Medical Sciences, Mashhad, Iran; Orthopedic Research Center, Mashhad University of Medical Sciences, Mashhad, Iran; Nurse, Mashhad Ghaem hospital, Ward of NICU, Mashhad University of Medical Sciences, Mashhad, Iran; Department of Pediatrics, Faculty of Medicine, Mashhad University of Medical Sciences, Mashhad, Iran

**Keywords:** Breastfeeding, dehydration, hypernatremia, lactation, lactation disorders, mother, neonate/newborn, pregnancy complications, risk factors

## Abstract

**Background:**

Neonatal hypernatremic dehydration (NHD) is a severe condition associated with neonatal morbidity and mortality.

**Purpose:**

The present study evaluated maternal risk factors, including duration of maternal hospitalisation, primiparity, caesarean section, and pregnancy complications, as well as social factors, such as depression, fatigue, and inadequate support for NHD.

**Data Sources:**

PubMed, Cochrane Library, International Scientific Indexing, Scopus, and Google Scholar were the databases searched until 2023.

**Study Selection:**

Articles written in English or Persian focusing on the relationship between maternal risk factors and NHD among neonates and providing sufficient information on NHD were included in this study. On the other hand, articles whose abstracts were only available were excluded.

**Data Extraction:**

The extracted data were presented in Excel software with the following titles: authors' names, year, type of study, study location, and maternal risk factors. The methodological quality of the articles was determined using the quality assurance tool for the diagnostic accuracy score (QUADAS).

**Results:**

Of the 58 searched articles, 16 were investigated, which included five prospective, seven cross-sectional, and four retrospective articles. Maternal risk factors for NHD included labour and delivery complications, childbirth complications, factors causing insufficient breast milk intake (including breast milk insufficiency, nipple problems, wrong breastfeeding techniques, breast disorders, types of feeding, and breastfeeding training/counselling in pregnancy), as well as delivery and the postpartum period.

**Implications for Practice and Research:**

Maternal problems in pregnancy and delivery, breast disorders, breastfeeding status, maternal knowledge, and lactation skills are the most common maternal risk factors for NHD. Timely (antenatal) identification and proper management of maternal risk factors help reduce the incidence and severity of NHD complications.

## Background and Significance

Neonatal hypernatremia is a condition in which serum sodium levels surpass 150 meq/L (1). Hypernatremic dehydration is associated with severe complications and can cause neonatal mortality in cases of delayed diagnosis. Therefore, healthcare providers are highly responsible for the teaching of appropriate breastfeeding techniques to mothers, as well as the early diagnosis and treatment of neonatal hypernatremic dehydration (NHD) (2). Hypernatremia is common during the first week after birth, so it is crucial to pay attention to its warning signs, such as weight loss, urine volume reduction, jaundice, and restlessness. Consideration of NHD symptoms, in addition to the assessment of neonatal weight and serum sodium levels in suspected cases, can be effective in the early diagnosis of this condition and the reduction of its lethal complications (3).

The prevalence of NHD is about 2–6% (3, 4). The incidence of NHD has increased due to inadequate maternal training, as well as a lack of knowledge and experience about breastfeeding (5). Reduced duration of maternal hospitalisation (6), caesarean delivery, primiparity, inadequate breast development during pregnancy, breast disorders, nipple abnormalities, ineffective or inadequate breastfeeding, low-frequency lactation, improper breast filling, emptying before and after each breastfeeding, delayed first breastfeeding, and the use of supplements such as manna or sugar water are considered risk factors for neonatal hypernatremia and severe neonatal weight loss during the first week of birth (7, 8).

### Literature Review

A prospective study with breastfeeding frequencies of 10.2 and 7.6 times per day in control and case groups, respectively, suggested that breast problems and lactation difficulties could be associated with the incidence of NHD (5). Since NHD can be prevented, all mothers must be trained in maintaining proper breastfeeding techniques, such as staying in the correct breastfeeding position. Dehydration symptoms should also be explained to mothers and close relatives, and neonatal weight must be measured during a neonatal visit at 3–5 days of life. In addition, mothers should solely feed their infant breast milk during the first six months of life, even during the summer (4).

Another important factor is that mothers should be trained for the frequency of breastfeeding, the baby's signs of satiety, and the amount of urine output. The healthcare team should perform a weight test after breastfeeding, and breastmilk should be expressed to determine whether the milk is sufficient. Dehydration percentile curves should also be used to monitor whether weight loss is pathological or not. The present study systematically reviewed maternal risk factors for NHD.

## Methods

After an initial review of the articles, a list of maternal risk factors for NHD was prepared, which included lactation, breast disorders, and other factors for NHD. Afterward, articles assessing maternal risk factors were searched for in the Cochrane Library, International Scientific Indexing, PubMed, EMBASE, Scopus, and Google Scholar databases. The keywords were hypernatremic dehydration, risk factors, maternal, mother, prognosis, complications, breastfeeding challenges, lactation, neonate, as well as labour and delivery. Overall, 58 studies met the inclusion criteria and were organised in a separate library file in EndNote software.

The present systematic review was performed on works published in English or Persian, according to the Preferred Reporting Items for Systematic Reviews and Meta-Analyses (PRISMA) statement (9). Of the 58 articles, 20 duplicates were eliminated, and the remaining 38 were evaluated in terms of their title and abstract. The article selection was performed based on the following criteria: 1) the study population being neonates, 2) NHD being confirmed, 3) maternal risk factors being investigated in infants with NHD, 4) articles being written in English or Persian, and 5) the information on the NHD status being available and sufficient. On the other hand, case reports, articles not reviewing maternal risk factors, and articles that only had abstracts available were excluded from the study. Of the 38 articles, 20 were irrelevant and removed, and two were omitted due to incomplete data, a lack of full text, as well as the uncertainty of the type of study and target group. Finally, 16 articles related to the research topic remained for the analysis.

The data, including authors' names, year of publication, type and location of study, case and control groups, as well as maternal risk factors, were extracted from the full-text articles obtained from the above databases and presented in Excel software.

The methodological quality of the articles was determined using the quality assurance tool for diagnostic accuracy score (QUADAS). This tool consists of 14 questions with “yes,” “no,” and “unspecified” answers scored as 1, −1, and 0, respectively, where the maximum score is 14.

## Results

Of the 58 articles, 16 articles published between 2006 and 2023 with a pooled sample size of 4,741 were studied (Flowchart 1).

**Flowchart 1.**
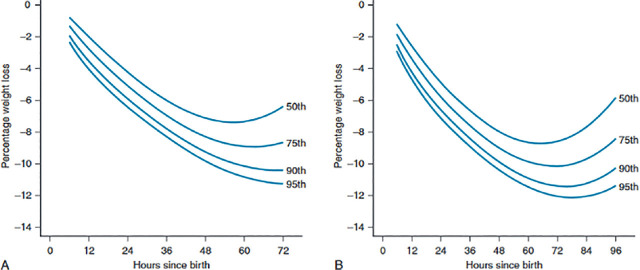
A, Estimated percentile curves of percent weight loss by time after birth for vaginal deliveries. B, Estimated percentile curves of percent weight loss by time after birth for caesarean deliveries.

The studies differed in their inclusion criteria, study population, case group definition, research methodology, sample size, and location. Of them, five were prospective, seven were cross-sectional, and four were retrospective (Table 1). The majority of the studies were carried out in Iran (7 [43.75%]), followed by Turkey (5 [31.25%]), India (2 [12.5%]), Sudan (1 [6.25%]), and Spain (1 [6.25%]). The investigated risk factors and the employed prevention methods for NHD are presented in Table 2.

**Table 1.** Summary of studies on maternal risk factors for neonatal hypernatremia

**No.**	**Author/year**	**Method**	**Location**	**Case group / Control group**	**Maternal risk factor**	**QUADA scores**
1	Boskabadi et al. (2014) (16)	Cross-sectional	Iran	670 cases	Breastfeeding frequency and status, let-down reflex, breast filling postpartum and after each lactation, breast softening after breastfeeding, duration of mother's admission	12
2	Boskabadi et al. (publishing)	Cross-sectional	Iran	934 neonates	Breast problems, lack of let-down reflex	13
3	Boskabadi et al. (2016) (3)	Descriptive - analytic	Iran	418 neonates	The time of first lactation, breastfeeding frequency	13
4	Uras et al. (2007) (62)	Retrospective	Turkey	1150 neonates	Breast problems	13
5	Boskabadi et al. (2013) (63)	Cross-sectional	Iran	273 neonates	Let-down reflex, mastitis, breast tightening before each lactation, breast softening after each feeding, breastfeeding status	12
6	Boskabadi et al. (2010) (6)	Prospective	Iran	106 neonates	Breast problems	13
7	Boskabadi et al. (2022) (2)	Cohort	Iran	183 neonates	Inappropriate breastfeeding	12
8	Mujawar et al. (2017) (19)	Retrospective	Iran	8 neonates	Breast milk sodium	13
9	Caglar et al. (2006) (8)	Prospective	Turkey	34 neonates	Breast problems with breastfeeding difficulties, Primipara, delay in theinitiation of first breastfeeding, cesarean section	13
10	Ramesh et al. (2017) (20)	Descriptive	India	201 neonates	Primipara	12
11	Goyal et al. (2018) (21)	Prospective	India	384 neonates	Cesarean section, Primipara, pregnancy complications, delay in the beginning of lactation after delivery, poor latch-on during breastfeeding, lack let-down reflex, decreased frequency of lactation, nipple difficulties	13
12	López Martín et al. (2018) (64)	Retrospective	España	20 neonates	Primiparity and greater maternal age	12
13	Orgunet al. (2019) (35)	Cross-sectional	Turkey	100 neonates	High breast milk sodium level and primiparity	13
14	Celik K (2021) (65)	Prospective	Turkey	47 neonates	Primiparity, insufficient breastfeeding	13
15	Alp EK (2022) (22)	Retrospective	Turkey	54 neonates	Insufficiency of breast milk, nipple problems, incorrect breastfeeding techniques	13
16	Zayed ME (23)	Cross-sectional	Sudan	206 neonates	Insufficiency of breast milk, breast problems	12

**Table 2.** Risk factors and prevention methods of neonatal hypernatremic dehydration

**Maternal risk factors**	**Prevention methods**
**Lactation Factors:** Maternal breastfeeding status, let-down reflex, breast filling after delivery and lactation, breast filling before each lactation, breast softening after lactation, delay in the onset of first lactation, reduced breastfeeding frequency, poor latch-on during breastfeeding, decreased or lack of mammary gland development during pregnancy, history of breast surgery, hypoplastic or abnormal breasts, endocrinopathies-induced infertility, pituitary dysfunction after delivery bleeding, retained placenta, delay in the onset of lactation for more than 12 to 24 hours postpartum, irregular breast drainage or stimulation, breast congestion or lack of breast enlargement after delivery, lack of colostrum production, technical problems of breastfeeding position, breastfeeding with one breast or early separation of the infant from the mother's breast during breastfeeding and before full drainage of breast milk, high breast milk sodium level. breast milk sodium level. **Breast factors:** Breast problems, lack of let-down reflex, mastitis, nipple difficulties. **Maternal factors:** Duration of maternal hospitalization, primiparity, greater maternal age, cesarean section, pregnancy complications, social factors such as depression, fatigue and inadequate support.	**Maternal factors:** attention to the frequency of breastfeeding and the situation of lactation, the elimination of breast problems and the improvement of milk production and transmission, more breast examination during prenatal and postnatal periods, encouraging to start lactation immediately after delivery and immediate intervention in case of breast feeding problems (poor latch-on, breast congestion, delayed milk outflow and nipple difficulties). **Neonatal factors:** check of neonatal weight and frequency of urination and defecation especially during the first week of life; feeding frequency more than 8 times per day.

Once lactation has commenced and is sustained, human milk production is governed by a blend of physical and biochemical factors. Regular milk removal is crucial to avert a rise in intermammary pressure and the accumulation of feedback inhibitors, which may result in reduced milk production and the onset of mammary involution. Conversely, consistent expression of breast milk eliminates these inhibitors, enabling the resumption of milk secretion. The production of human milk is regulated by a feedback loop that adjusts to the infant's consumption, guaranteeing alignment with the infant's requirements (10).

Various factors play a role in the duration of exclusive breastfeeding, including twin pregnancies, the mother's perception of insufficient breast milk, short maternity leave, the use of a pacifier for the infant, the infant's irritability, birthplace, and the presence of a homemaker. Accordingly, it is suggested that health policymakers provide more support programs for mothers with full-time jobs. Furthermore, more comprehensive educational programs should be designed to inform mothers of the signs of breast milk insufficiency and the barriers to exclusive breastfeeding, including pacifiers and sugar water (11).

Maternal breast problems with lactation complications, primiparity, delayed first lactation, delivery by caesarean section, and the use of warmers have been stated as risk factors for NHD (7). In cases of weight loss, the probability of maternal breast problems should be considered, lactation status should be assessed, and necessary interventions must be performed to eliminate breast difficulties and improve milk production and transfer to infants (7). Maternal diabetes-related factors, such as breastfeeding problems, may affect long-term lactation continuation in the early postpartum period (12). Breast problems, the lack of let-down reflex, and caesarean delivery have been reported to be less frequent among newborns, with a breastfeeding frequency of more than eight times a day (7).

The findings of the Boskabadi H study revealed that compared to caesarean delivery, normal vaginal delivery yields superior outcomes in terms of breast issues, breastfeeding success, labour duration, and length of maternal and infant hospital stays. Therefore, implementing meticulous guidelines in the management and conduct of deliveries could increase the likelihood of opting for normal vaginal delivery and enhance post-delivery recovery outcomes (13).

Mothers who received prenatal care, had a normal vaginal delivery, maintained proper breastfeeding positioning, and experienced a strong let-down reflex encountered fewer breast issues. Neonatal complications associated with breast problems include excessive weight loss and reduced frequency of urination. Consequently, closely monitoring the mother's breast health during pregnancy and immediately after delivery, along with administering suitable treatment, may help mitigate breast-related concerns and their impact on newborns (7, 14).

In a previous study, we reported primiparity and high maternal age as risk factors for neonatal hypernatremia (15). The assessment of newborns' weight, lactation frequency, breastfeeding position, breast changes during lactation, as well as urination and defecation frequency, may be effective in the early detection of milk intake reduction and the prevention of its complications (16).

Delayed initiation of breastfeeding and its low frequency have been observed more frequently in hypernatremic neonates (3). In a cohort study with a 36-month follow-up, inappropriate lactation positioning was reported as a maternal risk factor for 17.5% of abnormal outcomes in children (17). As a result, paying attention to lactation situations can reduce the incidence of NHD and its complications. Prasad et al. reported that during the first 24 h postpartum, most mothers breastfed their infants 2–3 h after delivery, as more than half of them had undergone caesarean section, and those with natural vaginal delivery (NVD) mainly experienced episiotomy. These factors cause severe pain and interfere with the onset of lactation (18). Additionally, Mujawar et al. (2017) revealed that maternal milk sodium level was highly correlated with neonatal hypernatremia (19). Ramesh et al. showed that hypernatremia was common in infants of primipara mothers (20). In Goyal et al.'s study, neonatal hypernatremia was present in 2.5% of caesarean sections and 0.8% of NVDs. Moreover, it was significantly associated with *primiparity*, pregnancy complications, delayed breastfeeding initiation after delivery, poor latch-on during lactation, lack of let-down reflex, decreased breastfeeding frequency, and nipple difficulties (21).

In the Alp EK. study, the causes of NHD were insufficient breast milk in 37 cases (68.5%), inability to breastfeed effectively due to nipple problems in 2 cases (3.7%), and incorrect breastfeeding techniques in 15 cases (27.7%). A positive correlation was also reported between the degree of dehydration, the percentage of weight loss, and the amount of serum sodium. Given that the most common cause of NHD is breast milk insufficiency, teaching breastfeeding to mothers before discharge from the hospital seems important in preventing this problem (22).

Zayed ME. et al. reported that there was insufficient breast milk in 86 (41.7%) infants with NHD, and there were breast problems in 9 (4.4%) (23).

Arora I. et al. found an incidence of 4.7% for NHD. Delayed initiation of breastfeeding, inadequate breastfeeding techniques, and maternal breast-related issues were significant contributors to NHD. Primiparous mothers were also found to be at a higher risk. Therefore, there is a need for targeted breastfeeding education and support for primiparous mothers. The study also reaffirmed the critical role of frequent breastfeeding with an effective duration and daily weight monitoring in the prevention of NHD (24). Livingstone VH et al. reported that maternal and infant risk factors consist of poor breastfeeding techniques, insufficiency of lactation after postpartum haemorrhage, and breastfeeding disorders with a cleft palate or ankyloglossia, which can interfere with breastfeeding and cause insufficient milk (25). In Oddie S et al.'s study, hypernatremia was mainly caused by unsuccessful breastfeeding (26). In Suliman OSM. et al.'s study, excessive weight loss, multiple pregnancies, low education level of the mother, and normal delivery were found to be risk factors associated with breastfeeding-related hypernatremia (27).

## Discussion

According to the results of this systematic review, maternal problems in pregnancy and delivery, breast disorders, lactation status, and the mother's knowledge and lactation skills are associated with the incidence of NHD.

Maternal factors, including the history of preterm delivery, multiple pregnancies, obstetric morbidity, gestational diabetes, urinary tract and vaginal infection, premature rupture of membranes, oligohydramnios, polyhydramnios, placenta previa, abruptio placentae, addiction, preeclampsia, and cerclage, cause preterm labour and breastfeeding disorders (28, 29, 30). A previous study showed that maternal diabetes-related factors, such as breastfeeding problems during the early postnatal period, could affect the long-term continuation of lactation (12). Breastfeeding initiation is a challenge for diabetic mothers due to the increased incidence of pregnancy and delivery complications, caesarean sections, instrumental labour, as well as increased neonatal complications, such as prematurity, respiratory distress, and poor sucking (31, 32). In addition, the lactation process is delayed in diabetic mothers (33). Given the increased incidence of postnatal hypoglycemia among newborns with diabetic mothers, the early onset of breastfeeding can increase glucose levels (34). High levels of sodium in breast milk are closely related to NHD, and being a primiparous mother appears to be a significant factor in the high sodium content in breast milk. All pregnant women, especially primiparous ones, should be educated about infant nutrition and neonatal dehydration. Healthcare providers should also emphasise the importance of frequent milking and increasing fluid intake, especially in the summer (35).

The type of delivery in neonatal hypernatremia has different role in studies. In Ghalehgolab's study, hypernatremia was more common and severe in infants born through NVD due to the early discharge of newborns after labour (36). In another study, however, the type of delivery played no important role in the occurrence of hypernatremia (37). The type of delivery may be a risk factor for NHD by affecting the time of breastfeeding initiation (38). Mothers need more help and support to initiate breastfeeding immediately after childbirth, as lactation initiation is more delayed in caesarean delivery. Mother–infant skin contact improves the onset of lactation and its continuity (39). Moreover, mother–baby skin-to-skin contact after birth has beneficial effects on breastfeeding and can increase the success rate and duration of the first lactation (40).

In addition, elective caesarean section may be associated with an increased incidence of preterm birth due to inappropriate estimation of gestational age. Premature infants born at 34–37 weeks of gestational age often present with sucking challenges and are more prone to a higher percentage of weight loss in the first days. All these factors have negative influences on breastfeeding initiation (41). Furthermore, if preterm infants admitted to the NICU are separated from their mothers for a period of time, it leads to improper maternal breast drainage and causes nipple fissures, painful lactation, and reduced breastfeeding (42). The results of the study by Flaherman VJ et al. showed that distinct weight loss patterns based on delivery mode were noticeable as early as 6 hours post-delivery and continued over time. Approximately 5% of infants born vaginally and over 10% of those delivered via caesarean had shed ≥10% of their birth weight within 48 hours of birth. By the 72-hour mark, more than a quarter of caesarean-born newborns had lost ≥10% of their initial weight (43).

Observing the proper breastfeeding position and how to stimulate the let-down reflex can lead to maximum neonatal milk intake with the fewest problems for mothers (44). Lactation insufficiency is a public health concern, as breast milk substitutes increase the risk of morbidity and mortality among infants in developing countries are the most common cause of malnutrition. The incidence of lactation insufficiency has been estimated to range from 23–63% during the first four months after delivery (45). Successful breastfeeding is unlikely for mothers with a history of breastfeeding problems in their previous pregnancies (17). In some studies, the most common clinical manifestation of NHD was jaundice, with poor lactation being one of its causes (46, 47). Breast problems exacerbate lactation problems and weight loss. On the other hand, infant problems in breastfeeding increase breast problems, causing a vicious cycle of breastfeeding problems (17).

Breastmilk provides the ideal nutrition for the infant, and exclusive breastfeeding is recommended for the first six months. Therefore, the mother's adequate milk production is critical, and early milk production has been shown to significantly affect milk production during the established lactation (48). Milk synthesis is not limited by the mother's capacity to synthesise milk but rather by the infant's appetite (49). A significant relationship was observed in a study between neonatal hypernatremia and frequency of breastfeeding (50). Therefore, the benefit of successful breastfeeding in the first week of life has become more eminent (17, 19).

Sugar water and flixweed consumption have been significantly associated with an inappropriate state of lactation, a lack of let-down reflex, and reduced breastfeeding frequency and duration (51). Giving traditional remedies, such as sugar water, camel thorn (botanical name: Alhagi Pseudalhagi), and flixweed (botanical name: Descurainia Sophia), to breastfed babies to reduce jaundice is prevalent in Iranian culture. During the first days of life, newborns may have insufficient breastfeeding for various reasons, or the mother may feel uncomfortable during lactation. Many families in Iran believe that these remedies effectively calm babies and reduce hyperbilirubinemia (52). The primary maternal risk factors associated with neonatal jaundice included prematurity, blood group incompatibilities, preeclampsia, hypertension, diabetes mellitus, vaginal bleeding, delivery complications (such as the method of delivery, labour-related injuries, home births, skin bruising, and cephalohematoma), maternal and community cultural beliefs (including the use of traditional remedies), breastfeeding challenges, and a decline in breastfeeding rates (53).

In the study by Boskabadi H et al., one-third of neonates with idiopathic hyperbilirubinemia experienced severe weight loss, and hyperbilirubinemia was more severe in this group. The average weight loss in neonates with severe hyperbilirubinemia (>20 mg/dl) was three times higher than that in those with moderate hyperbilirubinemia (<20 mg/dl) (54).

Increasing the frequency of breastfeeding, promoting accelerated weight gain, and encouraging more frequent defecation can help lessen the severity of neonatal hyperbilirubinemia. Therefore, providing breastfeeding education to mothers, with a focus on enhancing the frequency of breastfeeding, is a beneficial strategy to reduce the severity of hyperbilirubinemia in newborns (55).

NHD affects growth parameters and developmental milestones of children. Occasionally the child's weight gain was normalised by the end of first year of life; although developmental delay continued, its severity was reduced with age (14).

Delay in the referral of infants to a physician may exacerbate undiagnosed breastfeeding problems and lead to reduced milk intake complications, such as NHD. Increasing the knowledge of families, especially mothers, about the importance of weight loss and early referral may prevent NHD complications (56).

Sufficient information on breastfeeding helps reduce the risk of complications and lactation disorders and prolongs breastfeeding duration. Practical and visual methods in lactation training programs are very effective and paying attention to the mother's questions about breastfeeding is essential. The complete performance of lactation training should begin in the prenatal period (57). Common breastfeeding education is a part of standard pregnancy care and may be provided individually or in groups by healthcare professionals or consultants. The pregnancy period is a good opportunity to educate pregnant women and their families about breastfeeding benefits as they decide on their infant's feeding during pregnancy (58).

Severe neonatal weight loss or insufficient weight gain indicates a reduction in milk production or inadequate breast milk transmission (59). Therefore, the infant should be weighed whenever signs indicate inadequate feeding to identify high-risk infants and decrease dehydration severity (60).

Exclusively breastfed infants will feed every 2–3 h or on demand. There is usually a four-hour rest period during the night. Adequate breastfeeding depends on interlinking stages, such as natural breast growth, lactation onset, continuous production, and proper breast milk drainage. The last one depends on effective breastfeeding techniques and the normal milk outflow reflex. The total daily milk intake depends on breastfeeding frequency and duration (61).

Parents and grandparents' beliefs form part of our culture with certain societal values. The identification of subcultures and correcting them with appropriate methods can effectively promote hygiene and improve health. These beliefs are sometimes helpful but occasionally problematic. For instance, the belief in swaddling (a traditional practice of wrapping a baby up gently in a blanket to help them feel calm and sleep) has significantly contributed to the infant's adaptation to extra-uterine life; however, in excessive cases, it can cause many problems for the infant's health, such as dehydration, renal failure, and seizure, in the first several days (14).

Since maternal role in neonatal health through breastfeeding is vital and undeniable, NHD caused by maternal difficulties in lactation, lack of education, and breastfeeding skills, as well as breast problems, is one of the most preventable problems in the first two weeks of life. Identifying maternal risk factors and mitigating them before the occurrence of NHD significantly promote the infant's health and help successful lactation (3).

To the best of our knowledge, the current study is the first to systematically review maternal risk factors for NHD.

### Limitations

The limitations of this study include a possible failure to access all published articles and reports, a lack of accurate, applicable, and high-quality reports, a lack of clear and uniform criteria in the studies on NHD, a lack of identical definitions for case groups in the studies, methodological and procedural differences in the studies, and a lack of uniformity. It is recommended to conduct further meta-analysis studies for a more detailed and comprehensive examination of maternal risk factors in NHD.

### Conclusion

The most common maternal risk factors for NHD typically involve the let-down reflex being absent, low frequency of breastfeeding, insufficient breast filling prior to each feeding, lack of breast softening post-feeding, breast disorders, mastitis, delayed initiation of breastfeeding, maternal issues in pregnancy and delivery, maternal awareness, improper breastfeeding methods, lack of lactation proficiency, and primiparity.

Mothers should receive training on the frequency of breastfeeding, initiating lactation soon after birth, breastfeeding repeatedly (especially during the first weeks and at night), avoiding traditional supplements for infants (like sugar water and manna), and ensuring appropriate room temperature and suitable covering for newborns.

The healthcare team should provide comprehensive maternal care throughout pregnancy, lactation skills training, conducting breast examinations, and addressing any anatomical disorders. It is crucial to evaluate the mother's lactation status, offer consistent support and guidance during hospital admission and the first week postpartum. Educate mothers on the symptoms and signs of NHD, such as reduced breastfeeding frequency, weight loss, decreased urination and defecation, breast issues, and jaundice. Assess weight before and after breastfeeding, express breast milk to assess sufficiency, and monitor dehydration using percentile curves. Utilise percentile curves to track weight loss post-delivery for both vaginal and caesarean births.

### Highlight

Maternal problems in pregnancy and delivery, breast difficulties, breastfeeding status, maternal knowledge, and lactation skills, low breastfeeding frequency, lack of breast filling before each lactation, lack of breast softening after lactation, breast problems, mastitis, delayed first breastfeeding, lactation duration, maternal hospitalization duration, primiparity are the most common maternal risk factors for hypernatremic dehydration.

### Author Contribution Statement (CRediT Roles)

Boskabadi Hassan: Conceptualization, Resources, Data curation, Software, Formal Analysis, Supervision, Funding acquisition, Validation, Investigation, Visualization, Methodology, Writing – original draft, Project administration, Writing – review & editing. Rakhshanizadeh Forough: Writing – original draft, Project administration, Writing – review & editing. Ali Moradi: Data curation, Software, Formal Analysis, Validation, Investigation, Visualization, Methodology, Project administration, Writing – review & editing. Asal Ramezani: Conceptualization, Resources, Data curation, Investigation, Visualization, Methodology, Project administration. Maryam Zakerihamidi: Methodology, Writing – original draft, Project administration, Writing – review & editing.
